# Trivalent chromium supplementation ameliorates adjuvant induced rheumatoid arthritis through up-regulation of FOXP3 and decrease in synovial Cathepsin G expression

**DOI:** 10.1007/s10787-022-01025-8

**Published:** 2022-07-13

**Authors:** Sally S. Hassouna, Eman Sheta, Inass Zaki, Sahar A. Harby, Eman A. Allam

**Affiliations:** 1 Internal Medicine Department, Rheumatology and Immunology Unit, Faculty of Medicine, Alexandria University, Alexandria, Egypt; 2 Pathology department, Faculty of Medicine, Alexandria University, Alexandria, Egypt; 3 Clinical Pharmacology Department, Faculty of Medicine, Alexandria University, Alexandria, Egypt; 4 Medical Physiology Department, Faculty of Medicine, Alexandria University, Alexandria, Egypt

**Keywords:** Trivalent chromium, Rheumatoid arthritis, Prednisolone, FOXP3, Cathepsin G

## Abstract

**Background:**

Rheumatoid arthritis (RA) is a known debilitating autoimmune disease. Immune-suppressants that are used for disease treatment have serious side effects, therefore, trivalent chromium (Cr (III)); which has shown evidence of its influences on some inflammatory pathways and cytokines; was used in this study for the first time to be assessed for its therapeutic effect in RA rat model and was compared to prednisolone in a trial to find a treatment with lesser side effects.

**Methods:**

Adult male albino rats were randomly divided into four groups: normal, untreated RA, prednisolone treated RA (1.25 mg/kg/day) and Cr (III) treated RA groups (80 μg/kg/day), induction of RA was done by subcutaneous complete Freund adjuvant injection. Study duration was 4 weeks throughout which arthritis scoring and weight measurement were pursued. Histopathological examination and immunohistochemical FOXP3 assessment were done for joint biopsies. Serum inflammatory markers (interleukin 17, interleukin 10, CRP) and synovial erosive arthritis marker (Cathepsin G) were measured. HDL and non-HDL cholesterol were estimated as well.

**Results:**

Cr (III) treatment showed marked clinical and histopathological improvement, also astonishing anti-inflammatory effects (increase in FOXP3 expression and interleukin 10, with decrease in interleukin 17, CRP and synovial Cathepsin G) to the extent that Cr (III) effects on inflammation abolishment were comparable to that of prednisolone and even better at some aspects. Moreover, Cr (III) was protective from side effects, i.e., weight gain and dyslipidemia that were seen with prednisolone treatment.

**Conclusions:**

Cr (III) is promising in treating RA and it lacks some side effects of accustomed immune-modulatory agents including prednisolone. Further experimental studies and clinical trials should be held to see the efficacy of Cr (III) in different doses and to assess its long term side effects when used for rheumatoid arthritis and other autoimmune diseases treatment.

## Introduction

Rheumatoid arthritis (RA) is a known autoimmune disease. It affects joints and may have extra-articular manifestations. Pathogenesis of RA is still unknown but may be due to some genetic causes and environmental factors that affect immune system and attack joints accordingly (National Institute of Arthritis and Musculoskeletal and Skin Diseases (NIH) 2014; Majithia and Geraci 2007).

Forkhead box P3 (FOXP3) is a master regulator of regulatory T cells (Treg cells) development and function. CD4^+^Foxp3^+^ Treg cells suppress other immune cells but can also produce pro-inflammatory cytokines like IL17A (Du et al. 2014; Jung et al. 2017). T helper 17 (Th17) cells are CD4^+^ T cells functions by proinflammatory cytokines secretion and protection against microbial infections. Altered ratio between Foxp3^+^ Treg and Th17 cells has an important role in immune-related diseases pathogeneses including RA (Fontenot et al. 2005; Shalini et al. 2015; Robert and Miossec 2018). On the other hand, Treg cells bind to dendritic cells resulting in an increase in IL10 expression which is one of the anti-inflammatory cytokines that increases in RA remission (Greenhill et al. 2014).

Cathepsin G (CTG) is one of the serine proteases found in immune cells. There is evidence that CTG level increases in RA and is related to its immune-pathogenesis and joint destruction (Gao et al. 2018; Behl et al. 2022).

Treatment of RA aims to reduce inflammation and to improve patient’s sense of well-being and functioning. NSAIDs and steroids are frequently used to reduce symptoms, whereas disease-modifying anti-rheumatic drugs (DMARDs) like methotrexate and hydroxychloroquine aim to slow down disease progression (National Institute of Arthritis and Musculoskeletal and Skin Diseases (NIH) 2014) When these treatments are not effective alone, biological DMARDs may be used. Glucocorticoids are used as a bridging therapy with DMARDs (Singh et al. 2016; Hua et al. 2020). All these medications have several side effects (Rheumatoid 2020) giving a motive to search for a treatment with lesser side effects which is better to be a naturally occurring element inside human body and involved in biological processes, an element like trivalent chromium (Cr (III)) that is essential for sugar and lipid metabolism in humans (European Food Safety Authority 2014).

One study tested Cr (III) level in RA and it was lower in RA patients than normal subjects(Mohamed 2016) but the other studies done for this purpose showed non-significance (Hansson et al. 1975).

Cr (III) appeared to have important roles in immunity and inflammation summarized in the following examples. When going through Cr (III) mechanisms of improving insulin sensitivity, it is noticed that Cr (III) affects some inflammatory cytokines and immune pathways (Moradi et al. 2019; Hua et al. 2012; Jain et al. 2010; Hoffman et al. 2014), some of these pathways are related to FOXP3 expression (Ruan et al. 2012; Huber et al. 2008), inhibition or activation of which is found to be useful for RA remission (Makarov 2001; McHugh 2019). Moreover, Cr (III) appeared to treat some inflammatory conditions associated with diabetes, such as respiratory system inflammation (Kolahian et al. 2015).

Cr (III) effects on lipid metabolism lead to decrease in membrane cholesterol (Chen et al. 2006) which may cause decreased inflammatory response as a contrary to what was shown in Surls et al., who found that when membrane cholesterol increases T helper 1 response is enhanced (Surls et al. 2012). Also Cr (III) inhibits lipid peroxidation and has an antioxidant effect (Ueno et al. 1988; Cheng et al. 2004) which is needed for RA remission (Prescha et al. 2018).

Cr (III) appeared to have an antidepressant effect (Komorowski et al. 2012) through increasing serotonin secretion (Herr et al. 2017) which in turn modulates immunity as already known (Herr et al. 2017), this may give a benefit of treating depression accompanying RA or its disabilities, on the contrary of glucocorticoids which when used for the disease treatment can cause depression (Qin et al. 2019).

Cr (III) does not only have roles in innate immune system but also in humoral one. Lymphocytic proliferation was found to decrease when copper was added to Cr supplementation in Rhee et al. study (Rhee et al. 2002). Another study found that antibody production decreased, spleen weight lessened, splenocytes number and percentage of blood lymphocytes were reduced in Oreochromis mossambicus fish when exposed to either trivalent or hexavalent Cr (Arunkumar et al. 2000).

Even when dealing with Cr (III) drug interactions, aspirin and indomethacin increase the absorption of Cr (III) (https://www.webmd.com/vitamins/ai/ingredientmono-932/chromium), this may indicate that Cr(III) is engaged into the anti-inflammatory pathways elicited by those drugs and their mechanism of action.

Cr (III); as an insulin dependent glucose transporter antidiabetic; suppresses the effects of harmful serum lipids (Feng et al. 2019), on the other hand some immune-modulatory medications elevate blood sugar level and cause dyslipidemia with their consequences and complications (Penfornis and Kury-Paulin 2006). Preventing dyslipidemia would be protective from premature atherosclerosis associating RA (Arias de la Rosa et al. 2018).

Due to serious side effects of RA medications and due to what was mentioned about roles of Cr (III) in inflammation and its beneficial effects, it is necessary to give a chance for Cr (III) to be experimented in the disease.

Cr (III), would it work on disease relief, would it compensate for other immune-modulatory agents? Here is the question.

### Aim of the work

The aim of our study is to discover the impact of Cr (III) on RA activity, if it would decrease joint destruction, the possibility of its usage for disease treatment and also to compare its effect with that of glucocorticoid on alleviation of arthritis.

## Materials and methods

### Drugs and chemicals

Complete Freund adjuvant (CFA) (heat-killed *Mycobacterium butyricum* suspended in mineral oil) was purchased from sigma Ardlich, Egypt (Cat. No: F5881). Prednisolone was in the form of prednisolone sodium phosphate syrup (commercially named as Predsol syrup^@^, Borg Pharmaceutical industries, Egypt). Cr (III) was in the form of Cr (III) tablets (iHerb, Source naturals). Tablets were crushed and made as suspension in gum acacia solution to be given orally by intragastric gavage.

### Experimental animals

Approval of Faculty of Medicine, Alexandria University institutional animal ethics committee for all of the experiment procedures was obtained with: (IRB code: 00012098, FWA: No.: 00018699; International Council of Laboratory Animal science organization (ICLAS) membership. The serial number for registration was 0305207.

Thirty two albino male rats, weighing 120–140 g were purchased from Medical Physiology department animal research laboratory, Faculty of Medicine, Alexandria University, Egypt and were housed there in plastic cages (4 rats in each cage) at 23 ± 3 °C of stable humidity and rats were having free access to standard rodents’ food and tap water in a natural day and night cycle.

### Study design

One week was allowed for acclimatization to housing conditions for all study animals. Then, induction of RA was done in 24 rats by subcutaneous (SC) injection of 0.1 ml CFA into the plantar surface of the right hind paw. Another booster intra-dermal injection of 0.1 ml was given into the root of the tail on the same and on the following days (Darwish et al. 2013; Shirani et al. 2021). The remaining 8 rats, which served as the control group, received SC injection of an equal volume of mineral oil without any inflammation-inducing agent at the same anatomical sites. All injections were preceded by sterilization of skin with betadine antiseptic solution. The day of paw injection was considered day 0. On day 1, after appearance of local arthritis signs in the injected paw, CFA injected rats were randomly divided into three groups (8 rats each): untreated RA, prednisolone treated RA, and Cr (III) treated RA groups. Rats in the prednisolone treated RA group received low therapeutic dose of prednisolone (1.25 mg/kg/day). (Kuncha et al. 2014) Rats in the Cr (III) treated RA group received Cr (III) (80 μg/kg/day). (Komorowski et al. 2012; Chen et al. 2009; Sahin et al. 2013 Jul 28) Rats in the untreated RA group received gum acacia solution orally daily as placebo treatment. Treatment was given orally using a metallic gastric tube (gavage). Adequate measures were taken to minimize pain or discomfort. All treatments (prednisolone, Cr (III), and placebo) started from day 1 and continued till the study end at day 28.

### Clinical assessment and scoring of arthritis progression

Clinical assessment and scoring of arthritis progression were done at days 0, 1, 4, 8, 12, 16, 20, 24 and 28, depending on recording both the score of arthritis and the paw thickness. For arthritis score, paws were examined to see erythema and swelling severity by two of the experiment researchers and the score was given in the form of mean score for each paw.

A 5-point scale; mentioned in (Table 1); was used with maximum score of 4 for each paw. Only the three non-injected paws, which were having secondary arthritis, were scored with maximum arthritis score of 12 (Cremer et al. 1990), a score greater than 6 points indicates the presence of arthritis. Paw thickness was measured using a Vernier caliper on the same selected days to make an objective determination of foot inflammation and edema (nearly 0.02 mm) (Osada et al. 2009).

**Table 1:** Clinical and histopathologic arthritis scores

Clinical arthritis score	
0	No signs of inflammation
1	Mild swelling and erythema in the digit
2	Moderate swelling and erythema in the digit
3	Severe swelling and erythema in the whole area to the ankle
4	Severe swelling, erythema, disability and deformity of the limb
Histopathologic arthritis score	
*Intra articular exudate*	
0	No exudate
1	few inflammatory cells in joint cavity
2	Inflammatory cells partially filled joint cavity
3	Inflammatory cells totally filled joint cavity
*Synovitis*	
0	No inflammation
1	Mild
2	Moderate
3	Severe
*Cartilage erosion*	
0	Intact cartilaginous surface
1	Minor destruction in the cartilage surface
2	Focal loss of cartilage
3	Almost absent cartilaginous surface

### Weight measurement

All rats were weighed at days 0, 7, 14, 21, 28 and the weight in grams was plotted on a line chart. Weight measurement was used to assess severity of arthritis and effectiveness of treatments. Also weight measurement was used to calculate the appropriate dose of drugs.

### Study termination

Termination of the study was done on day 29. Rats were fasted overnight and anesthetized using ether inhalation then blood samples were obtained through cardiac puncture and after that animals were sacrificed. The collected blood samples were put in non-heparinized test tubes to undergo serum separation. Samples were centrifuged for 15 min at 3000 rpm. Separated serum was then stored at − 20 °C to be used for biochemical analysis. For histopathological examination the right hind paws were put in 10% formalin, while storage of the left ones was done at − 80 °C to be homogenized and then assessed biochemically.

### Histopathologic examination

Following amputation of the right hind paw of each animal above ankle joint the samples were sent to the Pathology Department, Faculty of Medicine, Alexandria University, and examined by two experienced pathologists. The samples that were fixed in 10% formalin for 24 h were cut longitudinally. Both halves were decalcified in EDTA solution and embedded in paraffin. Five microns sections were cut and mounted on glass slides. H&E stained section were examined without knowledge of sample label. At least three joints were assessed in each paw and the score was given according to the most severely affected joint (Larsson et al. 2004). Inflammatory changes in joints were graded according to the score listed in Table 1 (Caplazi et al. 2014).

### Immunohistochemical (IHC) staining for FOXP3

Five microns thick sections were cut and mounted on positively charged slides. Sections were deparaffinized and rehydrated in xylene and descending alcohol solutions, and rinsed with PBS. Sections were pretreated for antigen retrieval, and then stained with monoclonal antibodies against FOXP3 (Thermo Fisher Scientific, Cat No. 14-5773-82) with a dilution of 1:100. Staining was performed utilizing Envision detection system (Dako autostainer Link48) applying DAB as chromogen and hematoxylin as a counterstain. FOXP3 positive cells within inflammatory infiltrate were counted by recording positive cells per three high power fields using Image J 1.53e software. Then, the mean count per high power field was calculated (Eissa et al. 2016).

### Serum biochemical studies

#### Determination of serum levels of Interleukin 10 (IL10), IL17 and CRP

ELISA measurement of IL10 (MyBiosource Cat No. MBS2036296), IL17 (MyBiosource Cat No. MBS211293), and CRP (MyBiosource Cat No. MBS1754449) was done in the animals' serum samples. The analysis was done according to the manufacturer's protocol for commercial Kits.

#### Measurement of HDL and Non HDL cholesterol

Enzymatic methods were used to measure serum total and HDL cholesterol (HDL-C). Kits were obtained from BioSystems S.A., Costa Brava, 30. 08030 Barcelona, Spain and the procedure was done according to the manufacturer’s instructions. The following formula, (total cholesterol—HDL-C), was used to calculate non HDL cholesterol (Non HDLc). Atherogenic index was calculated according to the following formula: (Total cholesterol—HDL-C)/HDL-C (Basta et al. 2019).

#### Paw homogenization and determination of Cathepsin G (CTG) in paw tissues

The left hind frozen paw tissues were put in liquid nitrogen, then crushed and ground into powder by mortar and pestle. The paw tissues were then mixed with 100 mM of lysis buffer NaCl, 100 mM of EDTA, 0.5% Nonidet *p*-40, 0.5% Na-deoxycholate, Tris, pH 7.5 containing protease inhibitors cocktail (10 mM). The homogenate was then centrifuged for 10 min at 2000 g (4 °C) and then the supernatant was taken and stored at − 20 °C for measuring protein concentration using Lowry et al. method (Komorowski et al. 2012). CTG level was determined in the supernatants using rat (Cath-G) ELISA kit (MyBiosource Cat No. MBS722510) according to the manufacturer's protocol and the results were expressed as pg/mg tissue protein (Lowry et al. 1951).

### Statistical analysis

Data were analyzed using version 20.0 of IBM SPSS software package. (Armonk, NY: IBM Corp). Continuous data were tested by the Shapiro–Wilk test for normality. Distributed data was expressed as a range of minimum and maximum, mean, standard deviation and also median. The four studied groups were compared using ANOVA followed by Post Hoc test (Tukey) for comparison of pairwise. While the quantitative variables that were not normally distributed in different groups were compared using Kruskal–Wallis test followed by Post Hoc test or Dunn's for multiple comparisons test used for pairwise comparison. Obtained results significance was done by judgment at the 5% level.

## Results

### Arthritis induction, progression and the effects of treatments

**Fig. 1:**
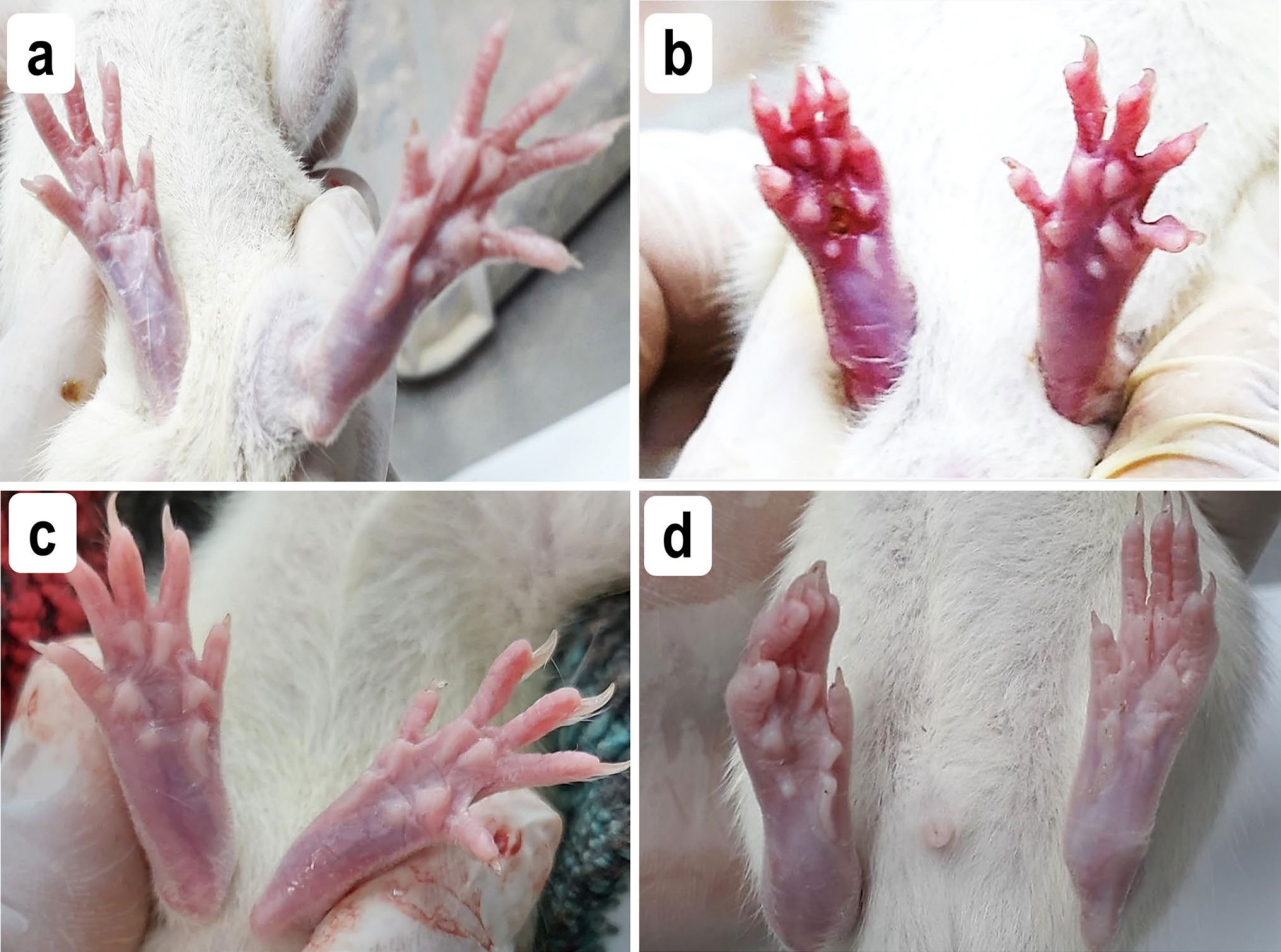
Representative photograph showing clinical signs of inflammation in rats’ joints examined at the end of the study. **a** Normal control rats, **b** untreated RA rats showing severe swelling and redness in both hind paws, **c** prednisolone treated RA rats, and **d** chromium treated RA rats. Less inflammatory signs are seen in both treated groups

All rats injected with CFA showed inflammatory signs in the injected right hind paw that were clearly apparent at day 1 and remained with variable degrees till the end of the experiment. These inflammatory signs were in the form of paw swelling, redness, and hotness with limitation of the paw movement, as was determined by foot dragging. The other non-injected paws exhibited similar inflammatory signs starting from day 12 till the end of the experiment. The course of these inflammatory signs was ascending with progression of arthritis in the untreated group. However, treatment either with prednisolone or Cr (III) was effective to alleviate these inflammatory signs in both treated groups. This was very evident by gross appearance as there was a great difference between the untreated and treated groups, as shown in Fig. 1.

### Clinical scoring of arthritis

**Fig. 2:**
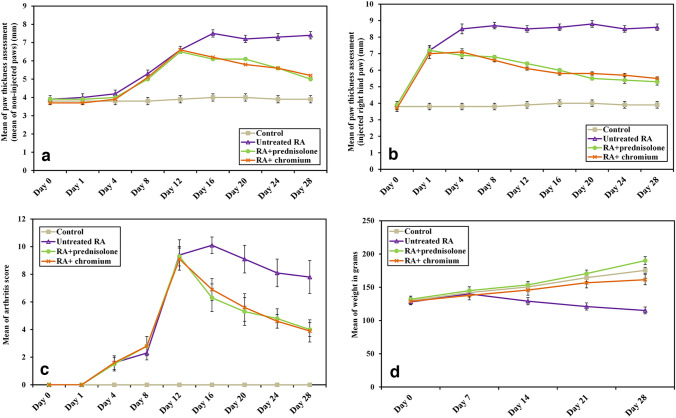
Effect of treatment with prednisolone or chromium on CFA induced changes in paw thickness, arthritis score and body weight. **a** Mean of thickness of non-injected paws, **b** thickness of injected paw, **c** arthritis score, and **d** body weight assessment

Assessment of paw thickness in the non-injected paws; assuming to have acquired secondary arthritis; showed that paw thickness in the control group differed significantly from the increased thickness in the three other groups, starting from day 12 and persisting throughout the remainder of the experiment (*P* =  < 0.001). However, there was an evident improvement and decrease in paw thickness in both treated groups either with prednisolone or Cr (III) which statistically differed from untreated RA group from day 16 and till the end of the experiment with a *P* value =  < 0.001. It is important to mention that there was no significant difference in paw thickness between both treated groups throughout the experiment (*P* = 0.826, 0.870, 0.102, 1.000 and 0.382 on days 12, 16, 20, 24 and 28, respectively; Fig. 2a). Nearly, the same results regarding the difference from the control group and the effectiveness of treatment can be applied on the thickness of the injected paw. However, changes in the injected paw thickness started from day 1 and persisted following the same course, as non-injected paws, till the end of the experiment (Fig. 2b).

The arthritis score done was reflecting both the changes in paw thickness and the presented inflammatory signs. On day 12, arthritis score in the control group differed significantly from the increased score in the three other groups till day 28 with a *P* value = 0.001. An improvement and decrease in the score with both treatments prednisolone and Cr (III) appeared on day 16 and was statistically different from untreated RA group and till the end of the experiment with a *P* value =  < 0.001. No significant difference in arthritis score appeared between both treated groups till the termination of the experiment (*P* = 0.988, 0.346, 0.674, 0.981, 0.988 on days 12, 16, 20, 24, and 28, respectively; Fig. 2c).

### Changes in body weight

At the start of the study, the weight of rats in all groups was in the same range without any significant difference. There was a significant decrease in body weight in untreated rats when compared to control group, this decrease started on day 14 and continued till the end of the experiment (*P* =  < 0.001). Weight observed in both treated groups was higher compared to that of the untreated one starting also from day 14 till the end of the study (*P* =  < 0.001). Weight in normal control group, on day 21, was significantly different from the decreased weight in Cr (III) treated group and the increased weight in prednisolone treated group with *P* value = 0.001 and *P* =  < 0.001 on day 28 for both (Fig. 2d).

### Joint histopathologic changes

**Fig. 3:**
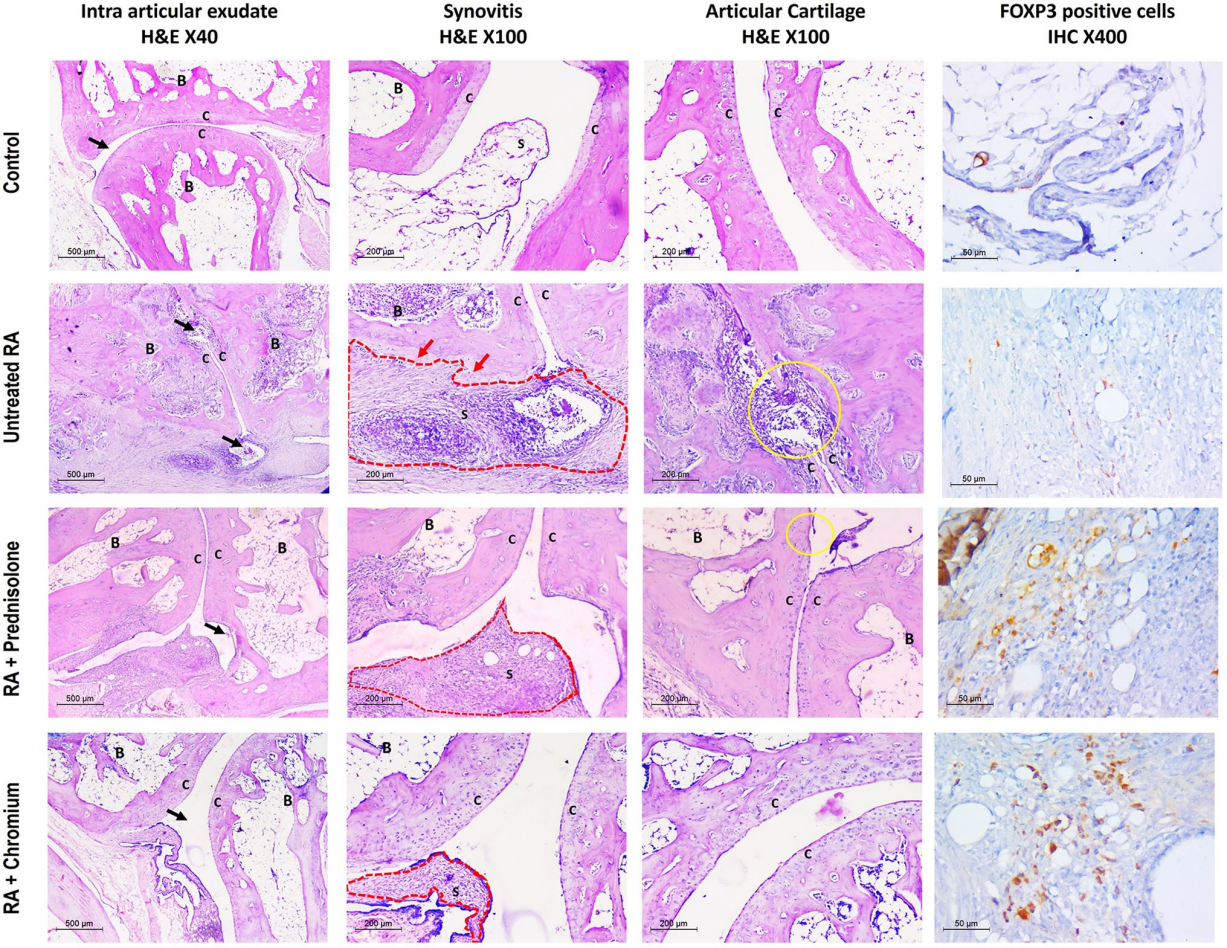
Effect of treatment with prednisolone or chromium on CFA induced changes in histopathology of hind paws; control: clear joint space (black arrow) with no synovitis or cartilage erosions. No FOXP3 positive cells are detected. Untreated RA: evident inflammatory changes are seen in the form of intra articular dense exudate (black arrows), severe synovitis (red dashed area) with pannus formation encroaching on cortical bone surface (red arrows) and focally lost cartilaginous surface (yellow circle). Few FOXP3 positive cells are seen. Prednisolone treated RA: moderate improvement is seen. Only few scattered inflammatory cells are seen in joint space (black arrow), moderate synovitis (red dashed area) with residual minor cartilaginous erosion (yellow circle) and moderate increase of FOXP3 positive cells. Chromium treated RA: marked improvement. No intra articular exudate (black arrow), mild synovitis (red dashed area) with restoration of cartilaginous articular surface and notable increase of FOXP3 positive cells. B = bone, C = cartilaginous articular surface, S = synovium, IHC = immunohistochemistry

Microscopic examination of control group revealed histologically free joints. The joint space was clear with no intra articular exudate. Synovial tissue was lined by a single layer of synoviocytes. No periarticular edema or inflammation. Articular surfaces were covered by intact layer of cartilage with normal subchondral bone histology. Untreated RA model showed polyarticular severe inflammatory changes. Intra articular exudate was noted in the form of mixed inflammatory infiltrate within joint spaces. Marked synovial thickening and synovial hyperplasia were detected. Dense heavy periarticular inflammatory infiltrate composed of numerous lymphoplasmacytic infiltrate and multinucleate giant cells was found with the formation of well-developed pannus. The latter invaded nearby bone in many areas. Cartilaginous articular surface was extensively eroded with focal total loss. Cr (III) treated RA group showed remarkable improvement of joint histopathology. Less synovial fluid cell infiltration was detected. Cartilage erosions and degree of synovitis varied greatly from untreated RA model. Cr (III) related changes were comparable to those seen in prednisolone treated group which showed residual edema and synovial thickening in some rats as well as minor cartilaginous erosions (Fig. 3).

**Fig. 4:**
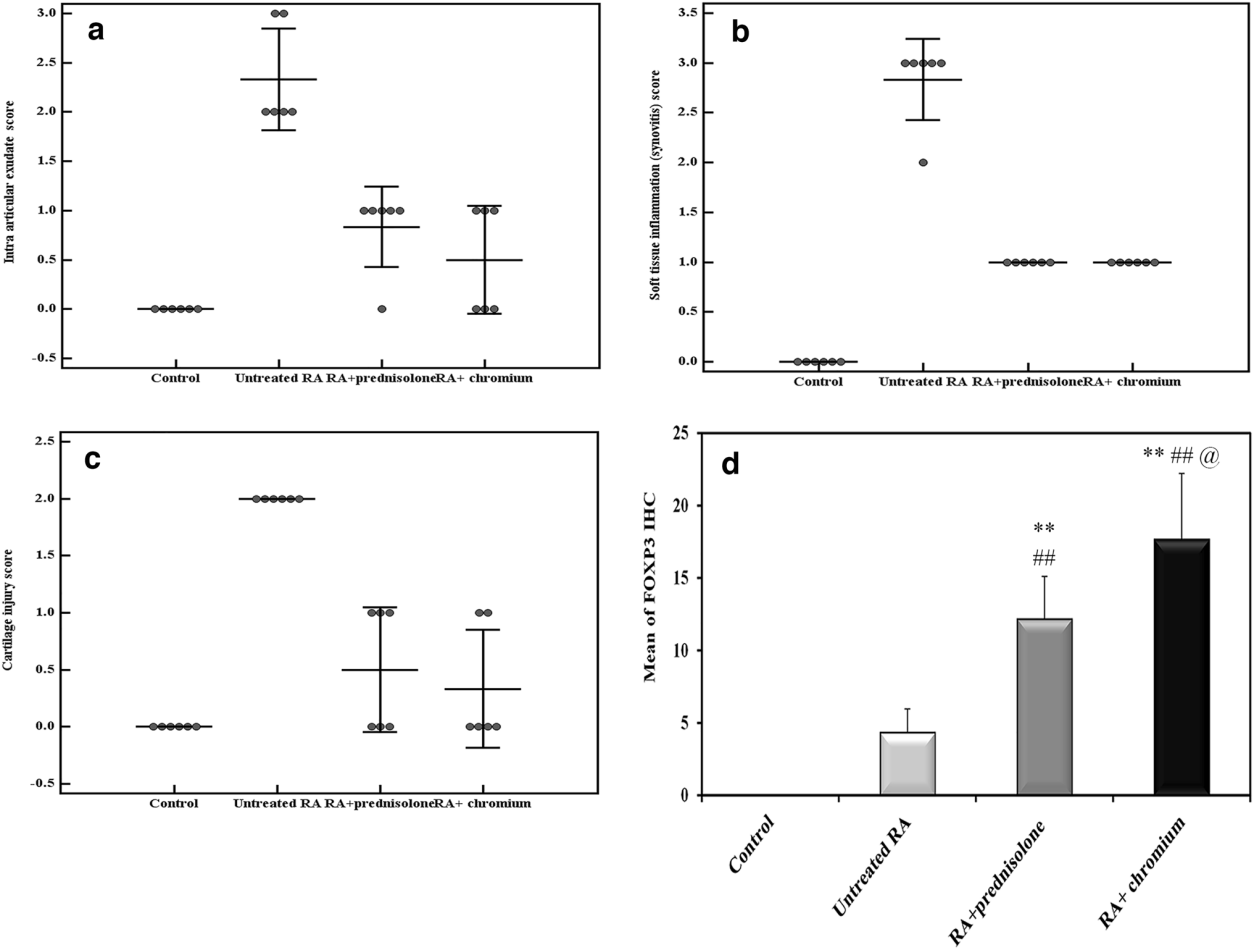
Effect of treatment with prednisolone or chromium on CFA induced changes in histopathology of hind paws. **a** Intra articular exudate, **b** synovitis, **c** cartilage erosions (three parameters are scored 0–3), and **d** number of FOXP3 positive cells by immunohistochemistry/HPF. **: Significantly different in comparison to control rats (*P* ≤ 0.001), ^##^significantly different in comparison to untreated RA rats (*P* ≤ 0.001), ^@^significant difference between both treated groups (*P* ≤ 0.05)

Histopathologic arthritis scoring for intra-articular exudate, synovitis and cartilage injury revealed that the three parameters were significantly increased in the untreated RA group when compared to the control group (*P* < 0.001 for the three parameters). Both treated groups showed significant improvement in the form of decrease in these scores when compared to the untreated RA group (*P* = 0.027 for exudate, *P* = 0.017 for synovitis and *P* = 0.007 for cartilage injury in the prednisolone treated group) and (*P* = 0.003 for exudate, *P* = 0.017 for synovitis and *P* = 0.002 for cartilage injury) in the Cr (III) treated group. However, no significant difference between Cr (III) and prednisolone treated groups regarding these scores was present (*P* = 0.435 for exudate, *P* = 1 for synovitis and *P* = 0.685 for cartilage injury). Both treated groups showed no significant difference with the normal control group regarding intra articular exudate (*P* = 0.051 for prednisolone treated group and *P* = 0.242 for Cr (III) treated group), also cartilage injury (*P* = 0.223 for prednisolone treated group and *P* = 0.416 for Cr (III) treated group). There were significant differences between both treated groups and its lower (zero) level in the control group only in synovitis with *P* = 0.017 for both treatments (Fig. 4).

### FOXP3 immunohistochemical expression

No FOXP3 expression was detected in control group, because no inflammatory infiltrate was present, meanwhile, RA untreated rats showed few FOXP3 positive cells (2–6/ HPF) among inflammatory infiltrate in periarticular tissues without a significant difference between both groups (*P* = 0.066). Both prednisolone and Cr (III) treated groups showed a notable increase of FOXP3 positive cell population (8–16/HPF and 13–25/HPF, respectively; Fig. 3). This was statistically significant in comparison of both with that of the control (*P* < 0.001 for both) and with that of the untreated RA group (*P* = 0.001 and < 0.001, respectively). Meanwhile, Cr (III) significantly increased the FOXP3 positive cells in comparison with prednisolone treated groups (*P* = 0.015; Fig. 4).

### Changes in the inflammatory markers (IL10, IL17 and CRP)

**Fig. 5:**
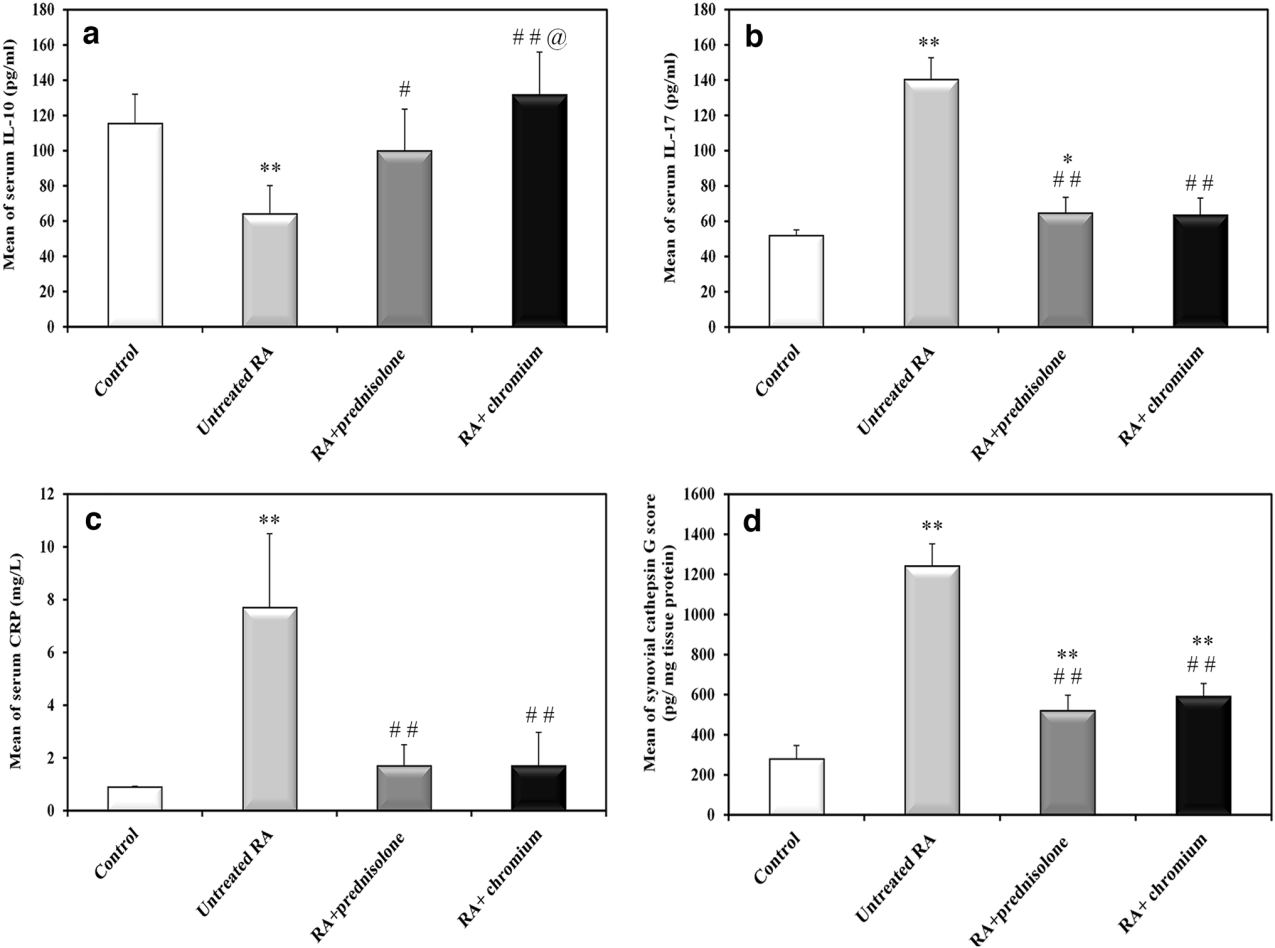
Effect of treatment with prednisolone or chromium on CFA induced changes in serum inflammatory markers and synovial erosive arthritis marker Cathepsin-G. **a** Serum IL-10, **b** serum IL-17, **c** serum CRP, and **d** synovial Cathepsin-G. *Significantly different in comparison to control rats (*P* ≤ 0.05), **significantly different in comparison to control rats (*P* ≤ 0.001), ^#^significantly different in comparison to untreated RA rats (*P* ≤ 0.05), ^##^significantly different in comparison to untreated RA rats (*P* ≤ 0.001), ^@^significant difference between both treated groups (*P* ≤ 0.05)

The anti-inflammatory cytokine IL10 was significantly decreased (44% decrease, *P* < 0.001), while the inflammatory markers IL17 (170% increase, *P* < 0.001) and CRP (7.5 folds increase, *P* < 0.001) increased in the untreated RA group versus the control group. Both types of treatment prednisolone and Cr (III) were effective to increase IL10 (56% increase, *P* = 0.008 in prednisolone group) & (105% increase, *P* < 0.001 in Cr (III) group) versus untreated rats. For IL17, it significantly decreased (54% decrease, *P* < 0.001 in prednisolone group) and (55% decrease, *P* < 0.001 in Cr (III) group) versus untreated rats. The same was applied on CRP, as both treatments significantly decreased its level (78% decrease, *P* < 0.001 in both treated groups) versus the untreated group. It is important to mention that all inflammatory markers returned to their control basal levels after both types of treatment except IL17 in prednisolone treated group it was still showing a significant increase versus its level in the control group (*P* = 0.043). Regarding the comparison between both treatment groups, no significant difference neither in IL17 nor CRP level was present (*P* = 0.995 for IL17 and *P* = 1 for CRP). However, Cr (III) treated group exhibited a significantly higher level of IL10 versus prednisolone treated group (*P* = 0.021; Fig. 5a–c).

### Changes in the synovial expression from the erosive arthritis marker Cathepsin G

Synovial CTG was up-regulated after RA induction in the untreated group versus the control group (3.4 folds increase, *P* < 0.001). Both prednisolone and Cr (III) were effective to produce significant 58% and 52% decreases, respectively, in comparison with the untreated group with *P* < 0.001 for both of them. However, CTG levels were still higher in both treated groups versus the control group with *P* < 0.001 for both of them. Both prednisolone and Cr (III) exhibited similar effects regarding their ability to decrease this erosive arthritis marker as no significant difference in its level was detected between both treated groups (*P* = 0.325; Fig. 5d).

### HDL and non HDL cholesterol

**Fig. 6:**
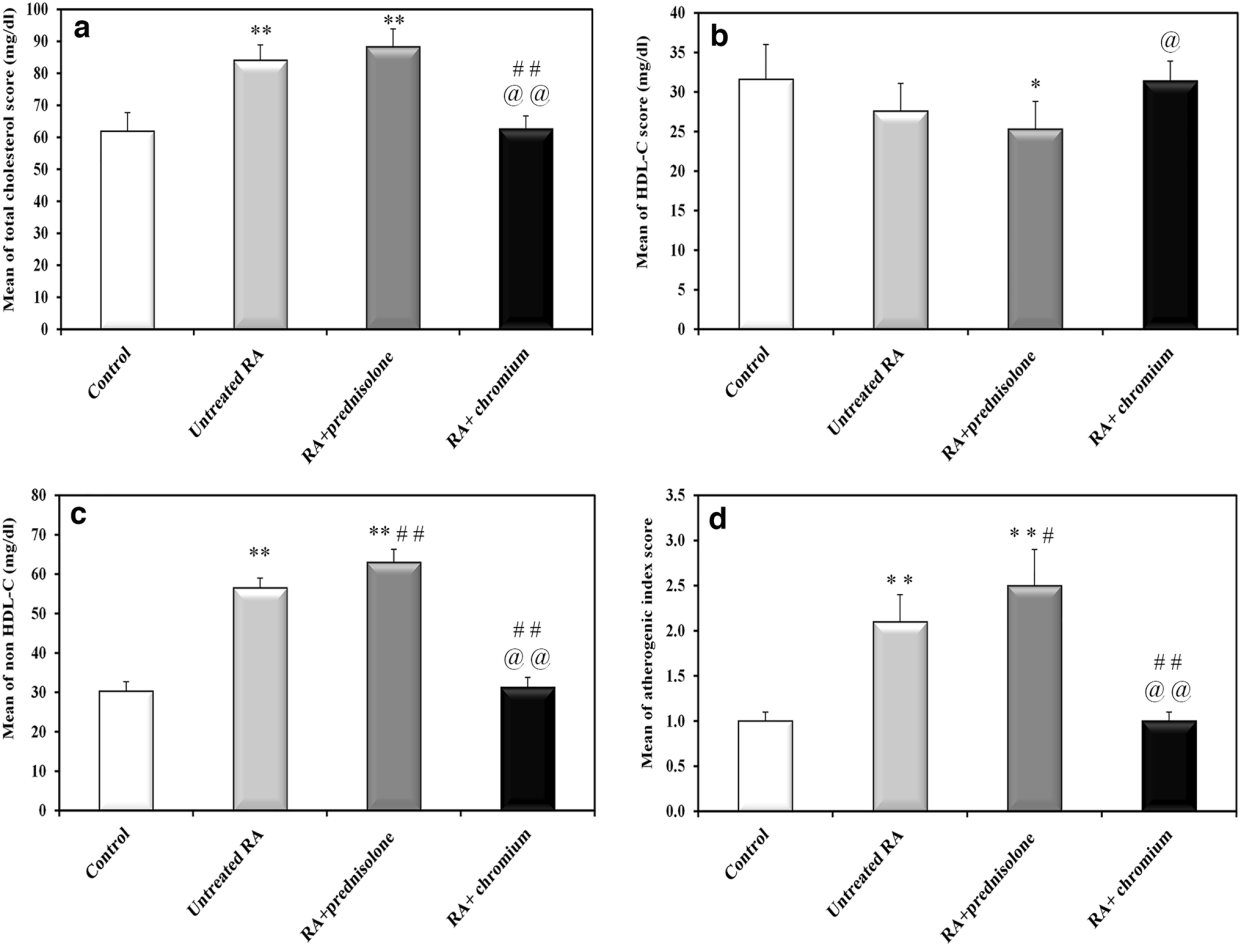
Effect of treatment with prednisolone or chromium on CFA induced changes in serum lipid profile. **a** Total cholesterol, **b** serum HDL-C, **c** serum non HDL-C, and **d** atherogenic index. *Significantly different in comparison to control rats (*P* ≤ 0.05), **significantly different in comparison to control rats (*P* ≤ 0.001), ^#^significantly different in comparison to untreated RA rats (*P* ≤ 0.05), ^##^significantly different in comparison to untreated RA rats (*P* ≤ 0.001), ^@^significant difference between both treated groups (*P* ≤ 0.05), ^@@^significant difference between both treated groups (*P* ≤ 0.001)

Untreated RA animals revealed significant increase in total cholesterol (36% increase, *P* < 0.001), Non HDL-C (86% increase, *P* < 0.001), and atherogenic index (110%, *P* < 0.001) versus those of control rats. While, HDL-C revealed 12.7% decrease in untreated RA group versus the control group but this decrease was not statistically significant (*P* = 0.136). Prednisolone treated RA rats exhibited increase in total cholesterol (5% increase), Non HDL-C (11.5% increase), atherogenic index (19% increase), and a decrease in HDL-C (8% decrease) versus untreated RA rats, the changes in Non HDL-C and atherogenic index versus the untreated rats were statistically significant (*P* < 0.001 and *P* = 0.002, respectively); however, the changes in total cholesterol and HDL-C were not statistically significant (*P* = 0.338 and *P* = 0.55, respectively. For prednisolone treated group it showed significantly higher total cholesterol, lower HDL-C and higher non HDL-C with (*P* values =  < 0.001, 0.007 and < 0.001, respectively, in comparison with normal control). Atherogenic index of prednisolone was significantly higher than normal control (*P* =  < 0.001; Fig. 6).

On the other hand Cr (III) treated RA rats revealed decrease in total cholesterol (26% decrease), Non HDL-C (45% decrease), atherogenic index (52% decrease), and an increase in HDL-C (14% increase) versus the untreated RA group. The changes occurred in total cholesterol, Non HDL-C and atherogenic index were statistically significant with *P* < 0.001 for all of them. However, the increase in HDL-C was not statistically significant versus the untreated group (*P* = 0.176). For Cr (III) treated group it showed no significant difference in total cholesterol, HDL-C and non HDL-C with (*P* values = 0.991, 0.999 and 0.879, respectively, in comparison to normal control). Atherogenic index of Cr (III) was not significantly different from normal control (*P* = 0.994). Cr (III) showed significantly lower total cholesterol, non HDL-C and atherogenic index than prednisolone (*P* =  < 0.001 for all), while HDL-C was significantly higher in Cr (III) treated group than prednisolone treated group (*P* = 0.009; Fig. 6).

### Toxicity study

Liver, kidneys, lungs and spleen biopsies were taken and microscopically examined in Cr (III) treated group and revealed normal histology with no pathological changes. Also liver and renal functions were done for this group and the results were within normal ranges.

## Discussion

RA is a chronic disease that may lead to disabilities and loss of function, most of the medications used to treat the disease have serious side effects, this is why there should be a search for an effective treatment with less side effects (National Institute of Arthritis and Musculoskeletal and Skin Diseases (NIH) 2014).

In the current study arthritis induction was successful and scored using arthritis scores. The dose of Cr (III) used was chosen in accordance with some studies which used the same dose of Cr (III) resulting in its anti-inflammatory effect (Chen et al. 2009; Sahin et al. 2013). Toxicity of Cr (III) was not reported when using higher doses than that used in the current study (Akhtar et al. 2020 Apr; White et al. 2021).

Untreated rats showed weight loss due to debilitation, while in treated groups rats were generally well and arthritis scores were better. Male was the chosen rats’ gender for the experiment, because engaging female rats may affect the inflammatory markers and biochemical investigations results due to their hormonal changes as evidenced in previous studies (Nunomura 1990; Arakawa et al. 2014).

This study results showed significant anti-inflammatory and anti-destructive effects of Cr (III) supplementations in experimental RA that appeared grossly, on clinical examination, on pathological assessment and in biochemical results, these effects may be explained by what was found in the study of Mohamed et al. who measured Cr (III) concentrations in plasma of RA patients and healthy control subjects and the results showed significantly lower level of Cr (III) in RA patients compared to healthy subjects(Mohamed 2016), this supports that Cr (III) deficiency may be a factor in RA pathogenesis; however, this was not proved in other studies, e.g., Hansson et al. who found no significant difference in Cr (III) level between RA patients and normal control subjects (Hansson et al. 1975).

This study revealed that Cr (III) up-regulates FOXP3 expression which means subsequent remission of RA (Kanjana et al. 2020) and this goes in line with the up-regulation of the anti-inflammatory cytokine IL 10 (Greenhill et al. 2014) in Cr (III) treated RA group that was even exceeding that of prednisolone in this study. IL 10 increase after Cr (III) supplementation was shown in the study of Odukanmi O. et, al who used Cr (III) in induced inflammatory colitis (Odukanmi et al. 2017). Cr (III) also showed up-regulatory effects on other anti-inflammatory cytokines (Moradi et al. 2019).

The increase in FOXP3 expression may also explain the decrease of IL17 after Cr (III) treatment in this study due to the imbalance of the Treg/TH17 cytokine axis found in RA pathogenesis (Al-Zifzaf et al. 2015). Decrease in IL 17 level appeared before in the American physiological society study (https://www.sciencedaily.com/releases/2010/09/100922155105.htm) which was investigating the effect of Cr (III) on diabetic nephropathy. Many other studies were done to show the effect of Cr (III) on other cytokines and found that it has an inhibitory action on other pro-inflammatory cytokines and decreases CRP such as what was shown in Jain et al. (2007) agreeing with this study results and also decreases HsCRP as shown in Zhang et al. (2021) Most of the studies done on Cr (III) effect on inflammatory cytokines showed that it has anti-inflammatory actions, only three human studies showed controversial results (Moradi et al. 2019).

For CTG, which decreased with both Cr (III) and Prednisolone treatments in this study, it has a crucial role in regulation of immune response and inflammation, also activates promatrix metalloproteinase-2 causing collagen gel contraction and leads to articular cartilage degradation in the cartilage–pannus junction of RA joints. Moreover, CTG appeared to be active and recruits monocytes inside synovial fluids of RA patients (Gao et al. 2018; Behl et al. 2022).

Weight loss which appeared in Cr (III) treated group in this study is beneficial for the wellbeing of the joints as this will decrease stress on them, this finding was opposite to what happened with prednisolone which is known to cause weight gain (Wung et al. 2008). Weight loss was mentioned in most of the studies that dealt with the effect of Cr (III) on body weight (Martin et al. 2006) but Maleki et al. found no effect of Cr (III) supplementation on body weight in polycystic ovary (Maleki et al. 2018).

The effect of Cr (III) on serum lipids that was encountered in the current study including decrease in non HDL cholesterol level and increase in HDL cholesterol level is agreeing with some studies talking about this point (Jain et al. 2006) while Balk et al. found that there is no effect of Cr (III) on serum lipids of non-diabetic subjects (Balk et al. 2007) and Tarrahi et al found no effect of Cr (III) on LDL level but it lowered total cholesterol, triglycerides and vLDL (Tarrahi et al. 2021). Cr (III) would be beneficial for treating dyslipidemia present in RA that gives rise to premature atherosclerosis in the disease (Arias de la Rosa et al. 2018), as what happened when Cr (III) was tested for prevention of atherosclerosis in diabetes (Qi et al. 2020). In the current study Cr (III) also showed a low atherogenic index and was significantly lower than prednisolone index which gives another advantage for Cr (III) in comparison with prednisolone which is known to cause dyslipidemia (Meng et al. 2017).

Increased HDL cholesterol with Cr (III) in comparison with prednisolone that was shown in the current study is also in favor of Cr (III) as HDL cholesterol is the good cholesterol which prevents vascular complications (Berrougui et al. 2012). Some DMARDS also increase HDL cholesterol level in RA after treatment with them (Navarro-Millán et al. 2013).

Although Cr (III) supplementation may be useful in RA patients, taking high doses of this heavy metal may have side effects like renal dysfunction and hepatotoxicity (Offenbacher et al. 1985; Wasser et al. 1997; Wani et al. 2006). However, the current study showed normal liver and kidney biopsies, liver and renal functions among Cr (III) treated RA. One of the suspected side effects of Cr (III) is genotoxicity which is said that it may happen with large doses but this is still under investigation (Sawicka et al. 2021; Eastmond et al. 2008).

## Conclusions

Cr (III) successfully treated induced RA with results comparable to prednisolone or even exceeding at some points. Cr (III); after further studies approving this finding; could be added to or substitute for immune-suppressive agents used for treatment of the disease or other autoimmune diseases especially that its benefits may overweigh its long term side effects on the contrary of other immune-suppressants.

## Data Availability

Enquiries about data availability should be directed to the authors.

## Abbreviations

RA: Rheumatoid arthritis
Cr (III): Trivalent chromium
FOXP3: Forkhead box P3
Th 17: T helper 17 cells
IL: Interleukin
CTG: Cathepsin G
DMARDS: Disease-modifying anti-rheumatic drugs
